# A Study of risk factors of postoperative ileus after laparoscopic colorectal resection

**DOI:** 10.1002/ags3.12705

**Published:** 2023-06-03

**Authors:** Sunao Fujiyoshi, Shigenori Homma, Tadashi Yoshida, Nobuki Ichikawa, Kengo Shibata, Hiroki Matsui, Akinobu Taketomi

**Affiliations:** ^1^ Department of Gastroenterological Surgery I Hokkaido University Graduate School of Medicine Sapporo Japan

**Keywords:** chronic obstructive pulmonary disease, colorectal cancer, laparoscopic surgery, postoperative ileus, risk factor

## Abstract

**Aim:**

Postoperative ileus (POI) is a common complication after abdominal surgery. However, the risk factors for POI after laparoscopic colorectal resection are unclear. We therefore investigated the risk factors for POI after laparoscopic colorectal surgery.

**Methods:**

This retrospective study involved 484 patients who underwent laparoscopic surgery for primary colorectal cancer at Hokkaido University Hospital. We categorized the patients into a POI group (*n* = 19) and non‐POI group (*n* = 465). We compared sex, age, smoking, chronic obstructive pulmonary disease (COPD), diabetes mellitus, body mass index (BMI), cardiac disorder, serum albumin, American Society of Anesthesiologists‐physical status, tumor location, tumor stage, operative duration, stoma formation, lymph node dissection, operator, and bleeding as potential risk factors for POI between the POI group and non‐POI group by univariate and multivariate analyses.

**Results:**

The univariate analysis results showed that the POI group had a higher incidence of male sex (*P =* 0.036), COPD (*P =* 0.029), and a BMI of <20 kg/m^2^ (*P =* 0.0487) as well as a higher bleeding volume (*P =* 0.014). The multivariate analysis results showed that male sex (odds ratio [OR], 0.2799; 95% confidence interval [CI], 0.089–0.993; *P =* 0.0298), COPD (0.2866; 0.095–0.862; *P =* 0.0262), and a BMI of <20 kg/m^2^ (0.2985; 0.112–0.794; *P =* 0.0154) were independent risk factors for POI after laparoscopic colorectal resection.

**Conclusion:**

Our findings suggest that male sex, COPD, and a BMI of <20 kg/m^2^ are independent risk factors for POI after laparoscopic colorectal surgery for treatment of colorectal cancer.

## Graphical abstract

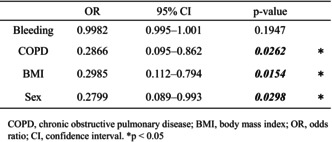

## 1 INTRODUCTION

Postoperative ileus (POI) is a common complication after abdominal surgery, and its reported incidence ranges from 10% to 17%.^1^ Most patients with POI improve with conservative medical treatments, but POI prolongs some patients' admission period and fasting time and adversely affects their activities of daily living and swallowing function. These problems place a medical financial burden on patients. Many risk factors for POI after colorectal surgery have been reported, such as male sex, previous abdominal surgery, stoma formation, intra‐abdominal infection, operation time, and laparotomy.^1^, ^2^, ^3^, ^4^ In recent years, laparoscopic surgery has become a mainstream technique in colorectal surgery (including robotic surgery), and we perform more than 90% of colorectal surgeries laparoscopically in our hospital. However, the risk factors for POI after laparoscopic colorectal resection are unclear. In this study we aimed to reveal the risk factors for POI after laparoscopic colorectal surgery.

## 2 METHODS

### 2.1 Patients

This retrospective study involved 484 patients who underwent laparoscopic surgery for treatment of primary colorectal cancer from January 2015 to December 2020 at Hokkaido University Hospital. Patients who underwent robotic surgery were excluded. We defined POI as Clavien–Dindo class <3a paralytic ileus. We categorized the patients to a POI group (*n* = 19) and a non‐POI group (*n* = 465).

### 2.2 Candidate risk factors for POI

We compared sex, age, smoking, chronic obstructive pulmonary disease (COPD), diabetes mellitus (DM), body mass index (BMI), cardiac disorder (including chronic heart failure, hypertension, valvular disease and ischemic heart disease), serum albumin, and operative duration, American Society of Anesthesiologists‐physical status (ASA‐PS), tumor location, stoma formation, lymph node dissection, tumor stage, operator, and bleeding as the candidate risk factors for POI between the POI group and non‐POI group. In a factor of operator, we defined “Resident” as physicians up to 5 y after graduation, and “Surgeon” as physician more than 5 y after graduation. Almost all “Surgeons” obtained endoscopic surgical skill qualification.

### 2.3 Statistical analysis

Sex, operator, COPD, DM, cardiac disorder, and stoma formation were compared using Fisher's exact test. Age, serum albumin, operative duration, years of smoking, and amount of bleeding were compared using the Mann–Whitney *U*‐test. ASA‐PS, tumor location, lymph node dissection, and tumor stage were compared using Pearson's chi‐square test. A receiver operating characteristic analysis was performed to determine the cutoff value for a high or low BMI. Using the cutoff value, the patients were separated into high and low BMI groups, and the BMI was compared using Fisher's exact test. Multivariate analysis was performed using logistic regression analysis.

All statistical analyses were performed using R software v. 3.4.0 (www.r‐project.org) and Bell Curve for Excel v. 2.00 for Windows (Social Survey Research Information, Singapore). *P* < 0.05 was considered statistically significant.

## 3 RESULTS

### 3.1 Patient characteristics

This study involved a total of 484 patients, and their characteristics are shown in Table 1. POI occurred in 19 (3.9%) patients, and no cases were more severe than Clavien–Dindo class 3b. The POI and non‐POI groups comprised 19 (3.9%) and 465 (96.1%) patients, respectively.

**TABLE 1:** Characteristics of patients (*n* = 484) treated by laparoscopic colorectal resection.

Sex	
Male	267 (55.2)
Female	217 (44.8)
Age, y	67.8 ± 11.6
Smoking, y	18.3 ± 19.7
COPD	
Present	47 (9.7)
Absent	437 (90.3)
DM	
Present	78 (16.1)
Absent	406 (83.9)
BMI, kg/m^2^	
<20	372 (76.9)
≥20	112 (23.1)
Cardiac disorder	
Present	204 (42.1)
Absent	280 (57.9)
Serum albumin, g/dL	4.1 ± 1.8
Duration, min	188.8 ± 66.9
ASA‐PS	
1	86 (17.8)
2	351 (72.5)
3	46 (9.5)
4	1 (0.2)
Tumor location	
Right colon	153 (31.6)
Middle colon	27 (5.6)
Left colon	88 (18.2)
Rectum colon	216 (44.6)
Stoma	
Present	79 (16.3)
Absent	405 (83.7)
Lymph node dissection	
D1	6 (2.3)
D2	124 (25.6)
D3	354 (73.2)
Tumor stage	
0	11 (2.3)
1	159 (32.9)
2	130 (26.9)
3	115 (23.8)
4	69 (24.2)
Operator	
Surgeon	447 (92.4)
Resident	37 (7.6)
Bleeding, mL	18.2 ± 85.4

*Note*: Data are presented as *n* (%) or mean ± standard deviation.

Abbreviations: ASA‐PS, American Society of Anesthesiologists‐physical status; BMI, body mass index; COPD, chronic obstructive pulmonary disease; DM, diabetes mellitus.

### 3.2 Determination of the cutoff value of BMI

We performed a receiver operating characteristic analysis of the POI group and non‐POI group to determine the cutoff value for a high and low BMI (Figure S1). The cutoff value of the BMI was determined to be 20 kg/m^2^.

### 3.3 Univariate regression analysis

We compared sex, age, smoking, COPD, DM, BMI, cardiac disorder, serum albumin, ASA‐PS, tumor location, and tumor stage as the patient factors and compared the operative duration, stoma formation, lymph node dissection, operator, and bleeding as the operative factors between the POI group and non‐POI group. The results showed a significantly higher incidence of male sex (*P =* 0.036), COPD (*P =* 0.029), BMI of <20 kg/m^2^ (*P =* 0.0487), and large amount of bleeding (*P =* 0.014) in the POI group (Tables 2 and 3). Therefore, these factors were further analyzed as possible risk factors for POI after laparoscopic colorectal resection.

**TABLE 2:** Univariate regression analysis of patient factors.

	Postoperative ileus		
	Present	Absent	
	19 (3.9)	465 (96.1)	*p* value
Sex			
Male	15 (78.9)	252 (54.2)	0.036*
Female	4 (21.1)	213 (45.8)	
Age, y	67.1 ± 13.1	67.9 ± 11.6	0.81
Smoking, y	21.6 ± 19.3	18.1 ± 19.7	0.32
COPD			
Present	5 (26.3)	42 (9.0)	0.029*
Absent	14 (73.7)	423 (91.0)	
DM			
Present	3 (15.8)	75 (16.1)	1.00
Absent	16 (84.2)	390 (83.9)	
BMI, kg/m^2^			
<20	8 (42.1)	101 (21.7)	0.049*
≥20	11 (57.9)	364 (78.3)	
Cardiac disorder			
Present	7 (36.8)	204 (42.4)	0.81
Absent	12 (63.2)	268 (57.6)	
Serum albumin, g/dL	4.1 ± 1.8	3.9 ± 0.55	0.34
ASA‐PS			
1	3 (15.8)	83 (17.9)	0.81
2	13 (68.4)	338 (72.7)	
3	3 (15.8)	43 (9.2)	
4	0 (0)	1 (0.2)	
Tumor location			
Right colon	6 (31.6)	147 (31.6)	0.99
Middle colon	1 (5.2)	26 (5.6)	
Left colon	3 (15.8)	85 (18.3)	
Rectum colon	9 (47.4)	207 (44.5)	
Tumor stage			
0	2 (10.5)	9 (1.9)	0.11
1	5 (26.3)	154 (33.1)	
2	3 (15.8)	127 (27.3)	
3	6 (31.6)	109 (23.4)	
4	3 (15.8)	66 (14.2)	

*Note*: Data are presented as *n* (%) or mean ± standard deviation.

Abbreviations: ASA‐PS, American Society of Anesthesiologists‐physical status; BMI, body mass index; COPD, chronic obstructive pulmonary disease; DM, diabetes mellitus.

* *P <* 0.05.

**TABLE 3:** Univariate regression analysis of operative factors.

	Postoperative ileus		
	Present	Absent	
	19 (3.9)	465 (96.1)	*P* value
Duration, min	192.8 ± 56.5	188.2 ± 67.3	0.4700
Stoma			
Present	5 (26.3)	74 (15.9)	0.22
Absent	14 (72.7)	391 (84.1)	
Lymph node dissection			
D1	1 (5.3)	5 (1.1)	0.12
D2	7 (36.8)	117 (25.2)	
D3	11 (57.9)	343 (73.8)	
Operator			
Surgeon	17 (89.5)	430 (92.5)	0.64
Resident	2 (10.5)	35 (7.5)	
Bleeding, mL	44.7 ± 106.4	17.2 ± 84.4	0.014*

*Note*: Data are presented as *n* (%) or mean ± standard deviation.

* *P <* 0.05.

### 3.4 Multivariate regression analysis

We performed a multivariate regression analysis of POI using sex, COPD, BMI, and amount of bleeding as potential risk factors. The results showed that male sex (odds ratio [OR], 0.2799; 95% confidence interval [CI], 0.089–0.993; *P =* 0.0298), presence of COPD (OR, 0.2866; 95% CI, 0.095–0.862; *P =* 0.0262), and a BMI of <20 kg/m^2^ (OR, 0.2985; 95% CI, 0.112–0.794; *P =* 0.0154) were independent risk factors for POI after laparoscopic colorectal resection (Table 4).

**TABLE 4:** Multivariate regression analysis of operative factors.

	OR	95% CI	*P* value
Bleeding	0.9982	0.995–1.001	0.1947
COPD	0.2866	0.095–0.862	0.0262*
BMI	0.2985	0.112–0.794	0.0154*
Sex	0.2799	0.089–0.993	0.0298*

Abbreviations: BMI, body mass index; CI, confidence interval; COPD, chronic obstructive pulmonary disease; OR, odds ratio.

* *P <* 0.05.

## 4 DISCUSSION

Millan et al^5^ reported that the risk factors for POI after colorectal surgery (including open and laparoscopic surgery) were male sex, an American Society of Anesthesiologists physical status of >3, current smoking, a serum albumin concentration of <3.5 g/dL, cardiac disorders, COPD, rectal tumors, performance of ileostomy or colostomy, and blood loss of >500 mL. Their study also suggested that there were no differences in the risk of POI between open surgery and laparoscopic surgery.^5^ However, there are few reports mentioned POI limited to patients undergoing laparoscopic surgery and no report mentioned COPD as the risk factor of POI limited to after laparoscopic colorectal surgery in colorectal cancer patients.

In this study we analyzed the risk factors for POI after laparoscopic curative resection. The results showed that male sex, a BMI of <20 kg/m^2^, and the presence of COPD were independent and significant risk factors. Some past studies showed that male sex was a risk factor for POI after colorectal resection.^1^, ^3^, ^5^ Our result regarding male sex was the same as that in past reports. In addition, our study suggested that a BMI of <20 kg/m^2^ and COPD were risk factors for POI after laparoscopic colorectal resection. We discuss the correlations of these factors with POI below.

### 4.1 Influences of hypercapnia and hypoxia on POI

COPD causes hypercapnia and hypoxia. Hypoxia can reportedly decrease intestinal peristalsis.^6^ Some past studies showed that high pneumoperitoneal pressure reduced blood flow to abdominal organs and that this could cause POI.^7^, ^8^, ^9^, ^10^ Furthermore, hypercapnia reportedly causes peripheral vessel shrinkage and organ ischemia.^11^, ^12^ These reports suggest that COPD can cause POI after laparoscopic colorectal surgery. In addition, Lee et al^13^ reported that a low BMI was related to subcutaneous emphysema and may therefore cause persistence of hypercapnia after surgery. These findings suggest that postoperative hypercapnia can cause POI and may explain why COPD and a low BMI were identified as risk factors for POI in this study.

### 4.2 Influence of nitrogen on POI

Nitrogen constitutes 75%–80% of gas in the dilated intestine due to ileus, and a decrease in the blood nitrogen partial pressure decreases the volume of intestinal gas.^14^ Nitric oxide (NO) suppresses intestinal peristalsis through the nervous system.^15^, ^16^, ^17^ In one study, surgical stress induced inflammatory mediators such as NO synthase (NOS) and cyclooxygenase‐2 in vascular endothelial cells, resulting in an increased serum NO concentration, and inhibition of these inflammatory mediators reduced the incidence of POI.^18^, ^19^ NOS is also produced and activated in lung component cells and inflammatory cells by cytokines and interleukins, resulting in the production of NO.^20^ In patients with COPD, however, it was found that NO production decreased by endothelial NOS because of endothelial dysfunction; as a result, the fractional exhaled NO concentration decreased and the serum nitrite level increased.^21^ These findings suggest that activation of vascular endothelial NOS and an increase in serum NO may occur in patients with COPD after laparoscopic surgery, physically and chemically contributing to POI.

Two specific measures may help to avoid POI in patients with COPD. First, maintaining a high level of oxygen in the perioperative period and introducing an antiinflammatory drug such as a cyclooxygenase‐2 inhibitor in the early postoperative period could be effective in the prophylaxis of POI. Second, hyperbaric oxygen (HBO) therapy may help to improve POI after laparoscopic surgery in patients with COPD. The mechanism by which HBO therapy improves POI is thought to occur as follows.

1. The increase in oxygen partial pressure and the decrease in nitrogen partial pressure promote the diffusion of intraintestinal nitrogen to the blood.
2. The increase in the blood oxygen level improves peripheral circulation and hypoxia in the intestine, resulting in improvement of intestinal peristalsis.

Thus, HBO therapy could be useful for preventing POI after laparoscopic surgery in patients with COPD because HBO therapy can counteract the increase in the partial pressure of carbon dioxide and nitrogen and the decrease in the partial pressure of oxygen, both of which contribute to POI after laparoscopic surgery. HBO therapy has also been reported as ineffective against POI.^22^ In addition, HBO therapy have a possible risk of pulmonary barotrauma^23^, ^24^ and hypoventilation under high concentration of oxygen for COPD patients. However, pulmonary barotrauma is very rare complication (0.00045%) in HBO therapy^25^ and the risk of barotrauma would vary, depending on the degree of COPD. Almost all cancer patients had a chest CT test to screen the lung metastasis and had spirometry to assess the respiratory function in the preoperative period. Therefore, we are able to evaluate the degree of COPD. We think that the mild COPD patients could undergo HBO therapy with low pressure, whereas severe COPD patients should undergo alternative treatment such as high flow nasal cannula or continuous low‐flow oxygen to prevent POI without the above complications. We believe that HBO therapy can effectively improve POI in some patients with mild COPD after laparoscopic surgery for the above‐stated reasons.

### 4.3 Limitations

This was a retrospective study in a single center, and the study population was relatively small. Therefore, we were unable to measure the blood oxygen level, carbon dioxide level, and nitrogen level of the patients in the perioperative period, and the impact of COPD on POI was only a matter of speculation from references. In addition, we have no data that measured the presence or absence of subcutaneous emphysema. In future studies, we should prospectively measure the blood gas levels in the perioperative period and the presence or absence of subcutaneous emphysema. In addition, a multicenter study is desirable to increase the number of patients.

## 5 CONCLUSION

Our findings suggest that male sex, COPD, and a BMI of <20 kg/m^2^ are independent risk factors for POI after laparoscopic surgery for colorectal cancer. These patients may be highly responsive to HBO therapy.

## AUTHOR CONTRIBUTIONS

Sunao Fujiyoshi designed the study, analyzed the data, and wrote the initial draft of the article. Shigenori Homma and Akinobu Taketomi contributed to the data analysis and interpretation and assisted in the preparation of the article. All other authors contributed to the data collection and interpretation and critically reviewed the article.

## FUNDING INFORMATION

No funding was received for this study.

## CONFLICT OF INTEREST STATEMENT

The authors declare no conflicts of interest for this article.

## ETHICS STATEMENTS

Approval of the research protocol: The Ethics Committees of Hokkaido University Hospital approved this study (no. 022‐0339), and informed consent was obtained from all patients by the opt‐out method in accordance with the guidelines of the Japanese Ministry of Health, Labour and Welfare (Tokyo, Japan).

Informed Consent: N/A.

Registry and the Registration No. of the study/trial: N/A.

Animal Studies: N/A.

## Supporting information


Figure S1.
