# Supplementary figures and images for: A Study of risk factors of postoperative ileus after laparoscopic colorectal resection
Source: Ann Gastroenterol Surg. 2023 Jun 3;7(6):949–54. doi: 10.1002/ags3.12705 (PMC10623944; doi:10.1002/ags3.12705)

Supplemental Figure S1

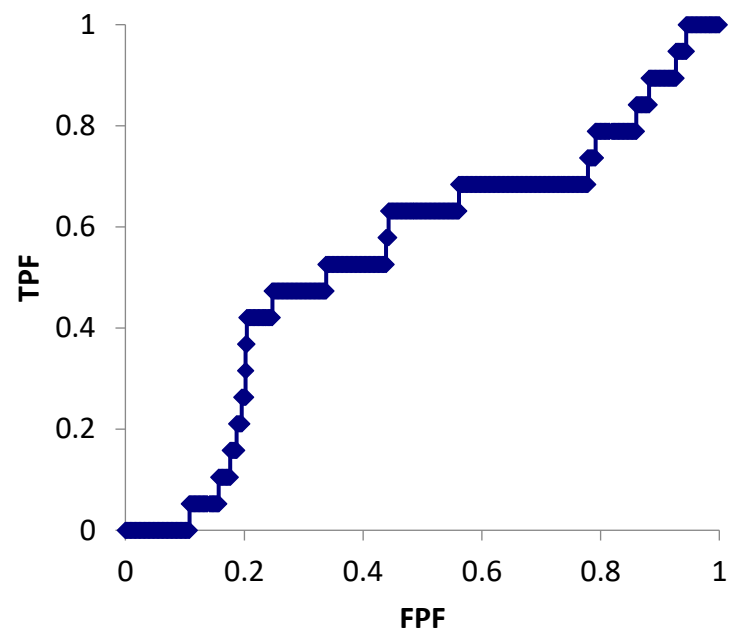

Supplement: Supplementary file 1 — Figure S1. [file AGS3-7-949-s001.pdf]
